# Current understanding and advances regarding the adipose-immune-metabolic axis in disease tolerance during sepsis

**DOI:** 10.3389/fimmu.2026.1755423

**Published:** 2026-03-03

**Authors:** Quanyue Du, Fanghao He, Shanchi Zhang, Xiongyan Lan, Yanqiu Pan, Jiajun Wang

**Affiliations:** Department of Critical Care Medicine, The Second People’s Hospital of Quzhou, Zhejiang University, Quzhou, China

**Keywords:** adipo-immune-metabolic axis, disease tolerance, lipid metabolism, lipolysis, sepsis

## Abstract

Sepsis remains a critical global health challenge characterized by high mortality and morbidity, primarily due to the limitations of current pathogen-centric therapies and a poor understanding of host-defense mechanisms. This review synthesizes the pivotal role of the adipose-immune-metabolic axis as a central regulator of disease tolerance—a host defense strategy that limits tissue damage without directly reducing pathogen load. We delineate how adipose tissue is reprogrammed from a passive energy reservoir into an active immunometabolic hub during sepsis. This functional shift is governed by three core hypotheses: “Metabolic Defense Priority,” which describes the preferential mobilization of fat to spare skeletal muscle protein; “Bidirectional Immunometabolic Crosstalk,” wherein immune cells such as macrophages and B cells precisely regulate lipolysis via specific cytokine signals (e.g., IL-1β and TGF-β); and “Stage-Specific Adaptation,” which outlines the dynamic evolution of axis function from the acute to chronic phases of sepsis. We further dissect key molecular pathways, including the Insulin-INSR-Thermogenesis, TGFβ-PDE3b-cAMP, and STING-ER Stress-mtROS axes, that orchestrate this complex interplay. Finally, we discuss contemporary challenges in mechanistic understanding, model translatability, and clinical translation, while proposing future directions to leverage this axis for developing novel, tolerance-based therapeutic strategies to improve sepsis outcomes.

## 1 Introduction

Sepsis, a life-threatening organ dysfunction caused by a dysregulated host response to infection, remains a critical global health challenge with persistently high mortality (1). While antimicrobial therapy is essential, the limitations of exclusively pathogen-centric approaches have underscored the urgent need to augment host resilience (2, 3).

This paradigm shift has brought “disease tolerance” to the forefront—a defense strategy that limits infection-induced tissue damage without directly reducing pathogen burden (4).

Central to the establishment of disease tolerance is the dynamic reprogramming of host metabolism and immunity (5). Emerging evidence now positions adipose tissue, far beyond a passive energy depot, as a pivotal immunometabolic hub that actively orchestrates this protective response during sepsis (6). It undergoes a profound functional shift, integrating systemic metabolic demands with local and systemic immune directives (7).

This review synthesizes current understanding of the adipose-immune-metabolic axis as a central regulator of disease tolerance in sepsis. We delineate its operational framework through three core hypotheses: “Metabolic Defense Priority,” “Bidirectional Immunometabolic Crosstalk,” and “Stage-Specific Adaptation.” Meanwhile we dissect key molecular pathways that underpin this axis, review contemporary challenges in research and translation, and propose future directions for leveraging this knowledge to develop novel therapeutic strategies aimed at improving sepsis outcomes. Furthermore, we examine how the function of this axis undergoes precise, stage-specific reprogramming across the acute, sub-acute, and chronic phases of sepsis, linking its adaptive capacity to clinical trajectories.

## 2 Adipose tissue and resident immune cells: an overview

### 2.1 Major types of adipose tissue and their functions

Adipose tissue is a dynamic endocrine and immunologically active organ (6), broadly categorized into white adipose tissue (WAT) and brown adipose tissue (BAT) adipose tissue, with an inducible beige phenotype. WAT, the primary site for energy storage, functions as a key endocrine organ by secreting adipokines (e.g., leptin, adiponectin) that regulate systemic metabolism and inflammation (8). Visceral WAT is metabolically more active and inflammatory, whereas subcutaneous WAT is more specialized in storage (9). In contrast, BAT is dedicated to thermoregulation via uncoupling protein 1 (UCP1)-mediated non-shivering thermogenesis, converting chemical energy directly into heat (10). This function is crucial for maintaining core body temperature.

### 2.2 The adipose immune microenvironment and its regulatory role

Adipose tissue houses a diverse population of resident immune cells, including macrophages, T cells, B cells, and eosinophils, which engage in constant crosstalk with adipocytes and stromal cells (11). Under homeostasis, anti-inflammatory macrophages (M2), regulatory T cells (Tregs), and eosinophils help maintain metabolic balance and insulin sensitivity (12, 13). This constitutive immunometabolic dialogue forms the foundation for adipose tissue’s capacity to undergo extensive functional reprogramming during stress (14). In sepsis, immune cells such as macrophages and B cells become critical regulators of adipose metabolism, precisely modulating processes like lipolysis through specific cytokine signals (e.g., IL-1β, TGF-β), thereby integrating immune responses with the host’s metabolic needs (15).

It is important to note that while foundational mechanistic insights discussed herein are largely derived from rodent models—which possess abundant and metabolically active BAT—the relevance and scale of these pathways in humans require careful translation. Key species differences, particularly in thermoregulatory strategies and basal metabolic rates, are addressed in detail in Section 5.2.

## 3 Theoretical background: core concepts and regulatory framework

### 3.1 Definition and characteristics of disease tolerance in sepsis

Disease tolerance in sepsis refers to a host defense strategy whereby the host activates a series of intrinsic physiological regulation and metabolic adaptation programs—without directly reducing the pathogen burden—to limit infection-induced tissue damage and organ dysfunction, thereby maintaining survival (16).Conventional defense strategies aim to recognize and eliminate pathogens, reducing pathogen load through mechanisms such as phagocytosis by immune cells, antibody neutralization, and inflammatory cytokines. In contrast, host organisms can also protect themselves from infectious diseases by mitigating the negative impact of infection on host fitness (17). Studies have revealed that sepsis survivors do not always exhibit lower pathogen loads than non-survivors, yet they consistently demonstrate better preservation of organ function (18). This critical observation provides a new therapeutic perspective: while administering appropriate antimicrobial therapy, we should simultaneously implement supportive measures to activate the host’s disease tolerance program. This involves using nutritional support, metabolic modulation, or pharmacological agents to enhance tissue resilience to ischemia, hypoxia, and inflammation, thereby protecting organ function and ultimately maximizing patient survival chances.

Disease tolerance mitigates infection-induced organ damage by optimizing the host’s physiological state—through metabolic reprogramming and inflammation buffering—without directly reducing the pathogen load. Metabolic reprogramming represents one of its core manifestations. Under infectious conditions such as sepsis, the host prioritizes the mobilization of triglycerides from adipose tissue, generating free fatty acids and ketone bodies via lipolysis to serve as primary energy substrates (19). The primary objective of this strategy is to minimize skeletal muscle protein catabolism. This is critical because sepsis potently activates proteolytic pathways, leading to rapid depletion of skeletal muscle—including respiratory muscles (20). The subsequent atrophy and functional impairment of these muscles, particularly the diaphragm (21),, constitute key factors contributing to ventilator dependence and mortality (22).Direct evidence demonstrates that dietary interventions which alter metabolic states can significantly enhance tolerance to diverse infections and improve survival rates, without affecting pathogen load (18). Inflammatory buffering does not entail completely shutting down the inflammatory response. Rather, it aims to reduce the pathology associated with the pathogen burden while actively promoting repair. During infection, the host’s immune response transitions from an early pro-inflammatory state to a later anti-inflammatory/repair phase. M2 macrophages, by secreting factors such as IL-10 and TGF-β, help suppress excessive inflammation and facilitate tissue repair (17).

In sepsis, disease tolerance serves as a critical host defense strategy. Its successful implementation is demonstrated by the optimization of core physiological states, with survival rate being the ultimate evaluation metric. This is specifically evidenced by: First, the maintenance of core body temperature, which relies on UCP1-mediated thermogenesis in brown adipose tissue. The functional integrity of this process is essential for preventing lethal hypothermia and improving survival (23). Second, the protection of organ function. Through mechanisms such as the suppression of the NLRP3 inflammasome by metabolites like ketone bodies, inflammatory tissue damage is mitigated. This can be objectively assessed by improvements in **Sequential Organ Failure Assessment (SOFA) score (**24) Third, the balancing of energy supply and demand. By activating a “metabolic defense priority” program, a lipolysis peak is reached during the sub-acute phase, preferentially mobilizing free fatty acids as the core energy substrates. This achieves protein sparing and safeguards organ function (18). The efficacy of these processes can be dynamically evaluated by monitoring the flux of metabolic substrates such as plasma free fatty acids and ketone bodies (7).

### 3.2 Architecture and functional role of the adipo-immune-metabolic axis

The Adipo-Immune-Metabolic Axis refers to a complex, bidirectional, and networked regulatory system formed among adipose tissue, the immune system, and systemic energy metabolism. It facilitates inter-cellular communication and functional synergy through direct cell contact, soluble factors (e.g., adipokines, cytokines), and metabolic intermediates, thereby collectively maintaining organismal homeostasis.

This axis is primarily composed of adipocytes, immune cells, and stromal vascular cells. Adipocytes are responsible for energy storage and release, as well as the secretion of adipokines. Adipose tissue is infiltrated by a multitude of immune cells—such as macrophages, T cells, B cells, and neutrophils—which engage in dynamic interactions with adipocytes. Stromal vascular cells, including preadipocytes and endothelial cells, also play critical roles in shaping the local microenvironment. Stromal vascular cells serve as a crucial platform for bidirectional immunometabolic crosstalk by maintaining adipose tissue structural integrity (25, 26). his ensures the efficient execution of lipolysis and ketogenesis central to the ‘Metabolic Defense Priority’ hypothesis (27), while simultaneously providing the microenvironmental support necessary for immune cell infiltration (28).

Adipose tissue is broadly categorized into two primary types: WAT and BAT. WAT is primarily recognized as an energy storage depot, but also functions as an endocrine organ, secreting various adipokines that influence systemic metabolic homeostasis. Notably, normal adipose tissue function becomes dysregulated in obesity. During sepsis, WAT undergoes lipolysis, breaking down stored triglycerides into free fatty acids and glycerol to provide emergency fuel for the body. Visceral white adipose tissue (vWAT) is metabolically more active and exhibits a stronger inflammatory response, whereas subcutaneous white adipose tissue (sWAT) is more specialized in energy storage. BAT is essential for thermoregulation through non-shivering thermogenesis, a key mechanism for heat production in mammals (10, 23). It severe metabolic stress of sepsis is postulated to activate this pathway, driving UCP1-mediated ‘futile cycling’ in BAT to generate heat. Maintaining normothermia is a critical challenge in sepsis, as dysregulated temperature profoundly impacts outcomes (29). Therefore, BAT-mediated thermogenesis may serve as an endogenous defense mechanism against sepsis-induced thermal dysregulation.

Immune cells—including innate immune cells (e.g., macrophages, neutrophils) and adaptive immune cells (e.g., T cells, B cells)—orchestrate adipose metabolism through the secretion of cytokines (e.g., IL-1β, TGF-β, IL-10) and surface molecule expression (e.g., CD73, PD-L1). Within the adipo-immune-metabolic axis, activated M1 macrophage infiltrate adipose tissue and secrete high levels of pro-inflammatory cytokines such as IL-1β and TNF-α. Specifically, macrophage-derived IL-1β activates the ERK signaling pathway within adipocytes, leading to direct phosphorylation and activation of hormone-sensitive lipase (HSL). This cascade drives a “non-canonical lipolysis” pathway that operates independently of the classical catecholamine-cAMP-PKA axis (30). B cells serve as critical negative regulators by secreting TGF-β, which upregulates phosphodiesterase 3B (PDE3B) in adipocytes. This enhanced PDE3B expression accelerates cAMP degradation, thereby suppressing hormone-sensitive lipase (HSL) activity and ultimately curtails lipolysis (31). Regulatory T cells and B cells contribute to the local adipose tissue microenvironment by expressing CD73 (ecto-5’-nucleotidase), which converts AMP to adenosine (32). The resulting adenosine acts on A2B receptors located on adipocytes, thereby inhibiting adenylate cyclase and reducing cAMP levels, which consequently leads to the direct suppression of lipolysis (33).

#### 3.2.1 Energy supply

During the early phase of sepsis, WAT undergoes intense lipolysis, driven by pro-inflammatory cytokines (e.g., TNF-α, IL-6) and stress hormones (e.g., catecholamines, cortisol) (7). This process is primarily orchestrated by the activation of HSL (34), resulting in a substantial release of released free fatty acids (FFAs) into the circulation. These FFAs not only serve as vital energy substrates but also function as signaling molecules that further modulate the activity of immune cells (35).

Under the synergistic drive of pro-inflammatory factors and stress hormones, white adipose tissue undergoes intense lipolysis. On one hand, cytokines such as TNF-α directly promote the activation of HSL by activating signaling pathways like ERK (36).these pathways lead to a sharp increase in plasma FFA levels (7).On the other hand, catecholamines phosphorylate and activate HSL via the classical β-adrenergic receptor-cAMP-PKA signaling axis, making this the rate-limiting step for lipolysis (34) The substantial amount of FFAs released not only serve as crucial energy substrates but also function as important signaling molecules. For instance, saturated fatty acids can further amplify the pro-inflammatory response by activating the TLR4 receptor on macrophages (35).

FFA released into the circulation are preferentially taken up by BAT, which is highly enriched with mitochondria. FFA undergo β-oxidation within BAT mitochondria, generating reducing equivalents that drive the electron transport chain (37) However, sepsis significantly induces and activates the unique UCP1 in BAT. Acting as a proton channel, UCP1 ‘uncouples’ oxidative phosphorylation, thereby diverting the chemical energy released from substrate oxidation away from ATP synthesis and converting it directly into heat (10). This nonshivering thermogenesis has been demonstrated in septic animal models to be a critical component of fever and the associated high energy expenditure (38).

Under the hypermetabolic conditions of sepsis, free fatty acids delivered to the liver not only fuel hepatocytes but are also partially converted into ketone bodies within hepatic mitochondria (19) The resulting ketones (e.g., β-hydroxybutyrate) serve as a highly efficient, water-soluble fuel that can be rapidly taken up and oxidized by the mitochondria of vital organs such as the heart and brain to generate ATP (39). The heart, in particular, utilizes ketone bodies as a preferred energy source, which helps sustain cardiac function under stress (40). Thus, the enhancement of ketogenesis during sepsis represents an evolutionarily conserved metabolic adaptation. It provides a critical alternative energy source for the body by converting stored fat into readily transportable and utilizable ketone bodies (41).

During the energy crisis of sepsis, the efficient utilization of the aforementioned lipid-based energy sources carries critical pathophysiological significance: namely, reducing the excessive reliance on skeletal muscle protein catabolism. It is well-established that during severe energy deficits, the body extensively breaks down muscle protein via the ubiquitin-proteasome pathway for energy production (20). Therefore, the active lipolysis, BAT thermogenesis, and hepatic ketogenesis observed in sepsis collectively constitute a metabolic adaptation strategy aimed at ‘protein sparing’. The essence of this strategy is disease tolerance—by prioritizing the mobilization of fat reserves to meet energy demands, the host maintains vital functions while maximizing the preservation of crucial structural and functional proteins (16). Substantial evidence indicates that, in the context of bacterial infections, interventions triggering this metabolic state (e.g., fasting) can significantly enhance host tolerance and improve survival rates through protein-sparing benefits (18).Furthermore, lipid metabolites such as ketone bodies may further contribute to tissue protection via direct anti-inflammatory mechanisms, among others (24).

#### 3.2.2 Immunological buffering

Mitochondria-Associated Endoplasmic Reticulum Membranes (MAMs) as Hubs for Inflammatory Sensing and Signal Integration, MAMs are dynamic, specialized membrane contact sites between the mitochondrial outer membrane and the endoplasmic reticulum, enriched with proteins mediating calcium signaling, lipid transport, and inflammatory responses (42, 43).During sepsis, this structure becomes a central platform for sensing both internal and external danger signals. On one hand, pathogen-associated molecular patterns (e.g., LPS) can transmit their downstream signals to the MAMs domain via TLR4 receptors on the surface of adipocytes and macrophages (44).On the other hand, host-derived damage-associated molecular patterns (e.g., mtDNA) generated by cellular stress can leak into the cytosol, be recognized by cGAS, and activate the STING pathway. The activated STING protein is precisely localized to MAMs to initiate a robust type I interferon response (45). Upon sensing inflammatory signals, MAMs remodel the immunometabolic microenvironment of adipose tissue. By mediating Ca²^+^ flux between the ER and mitochondria (46), MAMs directly regulate cellular metabolic states, promoting the production of a series of lipid mediators (e.g., PGE2) and metabolites (e.g., succinate) in adipocytes (47). This reprogrammed metabolic environment, particularly specific lipid species (e.g., palmitoleic acid) and signaling molecules, provides instructional cues for immune cell fate. Among these, prostaglandin E2 (PGE2) has been shown to directly drive macrophage polarization towards an M2 anti-inflammatory phenotype via the cAMP-PKA pathway (48). Concurrently, these metabolic changes help prevent the abnormal accumulation of metabolites like succinate, indirectly supporting the stabilization of the M2 phenotype (49). Ultimately, through the synergistic action of these metabolic signals and Th2 cytokines, macrophages differentiate into the classical M2 phenotype, characterized by high expression of arginase-1 and mannose receptors, and secretion of IL-10 and TGF-β (50). This establishes an anti-inflammatory and pro-repair microenvironment within adipose tissue.

Through the MAMs-STING axis, adipose tissue sensing of endogenous DAMPs (e.g., mtDNA) drives the polarization of macrophages toward an M2 phenotype (51). This shift establishes a local anti-inflammatory and pro-repair microenvironment (52, 53), which, by preserving adipose tissue homeostasis, systemically contributes to the maintenance of disease tolerance during sepsis (16).

### 3.3 The core theoretical hypothesis of adipo-immune-metabolic axis regulation in disease tolerance

#### 3.3.1 “Metabolic defense priority” hypothesis

Previous research has integrated the hypermetabolic phenomena observed in sepsis—such as lipolysis, ketogenesis, and BAT thermogenesis—into the “Metabolic Defense Priority” hypothesis (18). This hypothesis posits that the body prioritizes the consumption of renewable energy reserves (fat), even at a high energetic cost, to protect irreplaceable functional structures (muscle). Clinical observations directly validate the core metabolic features described by this hypothesis, demonstrating that septic patients exhibit significantly elevated plasma levels of FFAs and ketone bodies, the dynamics of which are closely associated with disease progression (7, 54).Furthermore, Baba et al. confirmed the activation of BAT in both rodent models and patients using imaging techniques (55),while Langley et al. revealed, through metabolomic studies, that survival outcomes are linked to specific metabolic states (54), Collectively, this clinical evidence provides robust support for the “Metabolic Defense Priority” hypothesis as a core host defense strategy.

These studies indicate that the metabolic phenomena observed during sepsis represent an evolutionarily conserved, active adaptive program. This ‘Metabolic Defense Priority’ program prioritizes the consumption of renewable fat reserves to spare structural muscle protein, aiming to maximize survival chances (18). It is executed through coordinated mechanisms: under the drive of inflammatory signals and catecholamines, white adipose tissue lipolysis releases FFAs into circulation (7, 56); a portion is converted by the liver into ketone bodies to provide an alternative fuel for the brain, reducing reliance on muscle catabolism (19); and another fraction is utilized by BAT for UCP1-mediated thermogenesis. By efficiently redirecting energy substrates through these pathways, the body delays sepsis-associated myopathy and preserves the function of vital muscles (57).

At the physiological level, the septic organism demonstrates the central role of fat mobilization and the achievement of protein sparing. At the clinical level, muscle loss has been consistently linked to adverse outcomes (57). At the experimental level, interventions that drive this metabolic switch directly enhance disease tolerance (18). This compelling body of evidence necessitates a paradigm shift in our understanding: the host is not a passive victim of infection, but rather actively orchestrates its energy resources through precise metabolic ‘switching’ (14).

#### 3.3.2 “Stage-specific adaptation” hypothesis

Sepsis is a dynamic, multi-stage pathological process. The “Stage-Specific Adaptation” hypothesis proposes that the disease tolerance strategy executed by the host via the adipo-immune-metabolic axis is not static, but undergoes precise, temporally ordered reprogramming as sepsis progresses (acute, sub-acute, and chronic phases).

##### 3.3.2.1 Acute phase (<no><18</no> hours)

This phase is characterized by the dominance of gluconeogenesis and muscle catabolism. The body prioritizes the fastest available energy pathways. Pro-inflammatory cytokines, along with high levels of glucocorticoids and catecholamines, dominate metabolism. Skeletal muscle protein breakdown is drastically activated via pathways such as the ubiquitin-proteasome system, serving as the primary source of amino acid precursors for gluconeogenesis (20).

##### 3.3.2.2 Sub-acute phase (18–72 hours)

Lipolysis and ketogenesis become the central metabolic processes. The inflammatory response becomes partially controlled, and bidirectional immunometabolic crosstalk becomes evident (56). A new balance is struck between pro-inflammatory signals and regulatory signals like TGF-β (18). Lipolysis in adipose tissue peaks during this stage, releasing free fatty acids that become the major energy substrates for organs like the heart and liver. Hepatic ketogenesis is significantly enhanced, providing ketone bodies as an efficient alternative fuel for the brain.

##### 3.3.2.3 Chronic phase (>72 hours)/PICS phase

This phase, also known as the Persistent Inflammation, Immunosuppression, and Catabolism Syndrome (PICS) stage, is marked by the failure of lipolysis and severe, uncontrolled protein wasting. Insulin resistance, growth hormone resistance, and dysfunction of the hypothalamic-pituitary axis emerge. Adipose tissue itself develops mitochondrial dysfunction and impaired insulin signaling. Hormone-sensitive lipase (HSL) activity is suppressed due to various factors, leading to impaired fat mobilization, reduced FFA release, and concurrent hypertriglyceridemia (resulting from increased hepatic lipogenesis and impaired clearance) (58). Consequently, adipose tissue—the body’s largest energy reservoir—becomes dysfunctional and fails to supply fuel effectively. The body is forced to revert to a reliance on the catastrophic breakdown of skeletal muscle protein for energy, creating a vicious cycle of “catabolism → wasting → functional loss,” leading to severe cachexia and multiple organ failure.

In sepsis, the metabolic fate of adipose tissue is largely determined directly by the infiltrating immune cell populations and their specific cytokine profiles. The capacity to mount and then resolve the adaptive metabolic and immune responses characteristic of the acute and sub-acute phases is a prerequisite for recovery. Conversely, the failure to resolve these responses and the subsequent descent into a state of chronic immunometabolic dysfunction (the PICS phase) is central to the progression toward persistent organ failure and poor outcomes (59).

The three hypotheses are hierarchically structured and mutually reinforcing, collectively forming a comprehensive theoretical framework for how the adipo-immune-metabolic axis governs disease tolerance. This framework is unified by the core objective of ‘protein sparing and organ protection,’ operationalized through bidirectional immunometabolic crosstalk to achieve precise fuel mobilization, and dynamically adapted across the distinct stages of the disease course.

## 4 Research landscape: key advances in understanding the adipo-immune-metabolic axis in regulating disease tolerance during sepsis

### 4.1 Core functions and mechanisms of adipose tissue in sepsis-induced disease tolerance

In the pathophysiology of sepsis, adipose tissue has evolved from a conventional energy storage organ into a dynamic and active immunometabolic hub.

#### 4.1.1 Energy supply: the role of adipose lipolysis and thermogenesis in disease tolerance

During the metabolic response to sepsis, the canonical lipolysis pathway serves as a crucial initial signal driving early energy mobilization. This pathway is primarily mediated by catecholamines via β-adrenergic receptors. Upon activation, the highly conserved signaling axis of Gs protein-adenylyl cyclase-cAMP- PKA is triggered, ultimately leading to the phosphorylation and activation of HSL, which hydrolyzes triglycerides to release FFAs (34).

Beyond classical endocrine regulation, adipose mobilization is also directly driven by non-canonical lipolytic pathways. These pathways are primarily activated by inflammatory signals (e.g., TNF-α, IL-1β) and operate independently of the classical β-adrenergic receptor-cAMP axis. Specifically, TNF-α exerts its effects by activating the JNK and ERK signaling pathways (60). The non-canonical lipolysis mediated by M1 macrophages represents the core driving force for the rapid energy mobilization from adipose tissue during early sepsis. A significant fate of the substantial amount of FFAs released via these lipolytic processes is their active uptake by BAT, facilitated by highly expressed fatty acid transporter proteins. Within BAT mitochondria, FFAs undergo β-oxidation; however, the resulting proton gradient is ‘short-circuited’ by UCP1. Consequently, the chemical energy is diverted away from ATP synthesis and is instead dissipated as heat (10). Furthermore, active BAT significantly accelerates the clearance of triglycerides and free fatty acids from the bloodstream. BAT thermogenesis, as a regulated energy expenditure pathway, safely dissipates the energy from excessively mobilized lipids in the form of heat, thereby mitigating lipotoxicity (61).

The products of lipolysis are not merely energy substrates but also crucial signaling molecules. Glycerol is transported to the liver, where it is catalyzed by glycerol kinase to serve as a key precursor for gluconeogenesis, helping to maintain blood glucose levels. Certain unsaturated fatty acids and their derivatives (e.g., oxidized fatty acids) can function as endogenous agonists for the nuclear receptor PPARγ. Binding and activation of PPARγ subsequently regulates anti-inflammatory gene expression programs, suppressing inflammatory responses in macrophages, among other cells (62). In contrast, saturated fatty acids (e.g., palmitic acid) can activate the TLR4 receptor, initiating pro-inflammatory signaling pathways such as NF-κB, thereby directly exacerbating the inflammatory response (63).

When the lipolysis-thermogenesis axis becomes dysfunctional, the host’s metabolic defense system collapses, leading to rapidly deteriorating outcomes.1.Lipolytic Failure: Chronic inflammation induces insulin resistance and receptor desensitization in adipocytes (64). Concurrently, inhibitory signals such as TGF-β persistently suppress cAMP levels by upregulating PDE3B, directly curbing lipolysis. Coupled with macrophage infiltration and intrinsic dysfunction within the adipose tissue itself (65), these factors collectively lead to insufficient FFA release. This interruption in energy substrate supply forces the body to revert to a reliance on the catastrophic breakdown of skeletal muscle protein, accelerating cachexia and organ failure (57).2. hermogenic Program Failure: Brown adipose tissue becomes functionally exhausted, manifesting as reduced mitochondrial density, downregulated UCP1 expression, and loss of the crucial protective role provided by Treg cells. Consequently, the body loses its capacity for thermogenesis, struggling to maintain core body temperature.3. Systemic Metabolic Dysregulation: Due to impaired utilization and suppressed lipoprotein lipase activity (7). the FFAs generated from lipolysis are re-esterified in the liver, leading to hypertriglyceridemia. This condition is not only a hallmark of defective lipolysis but can also exacerbate inflammation and organ injury by activating immune cells, thereby creating a vicious cycle (66).

During the subacute phase of sepsis, when the metabolic paradigm shifts toward lipid mobilization, a balanced and moderate level of *de novo* lipogenesis plays a crucial physiological role (67). Catalyzed by key enzymes such as acetyl-CoA carboxylase (ACC) and fatty acid synthase (FAS), adipose tissue can utilize substrates like glucose to synthesize fatty acids *de novo* and esterify them into triglycerides for storage. This process does not oppose lipolysis but rather complements it, helping to prevent premature depletion of adipose reserves and thereby maintaining a sustained and controlled release of FFAs—a stable energy substrate for vital organs such as the heart and skeletal muscle.

However, this delicate equilibrium is highly vulnerable to disruption. Progressive insulin resistance impairs the normal stimulatory effect of insulin on lipogenesis (5), while persistent inflammatory signaling—exemplified by cytokines such as TNF-α—directly suppresses the expression and activity of ACC and FAS, thereby potently inhibiting lipid synthesis (68). When lipogenesis is severely impaired in the face of ongoing lipolytic demand, adipose tissue fails to adequately replenish its triglyceride stores, leading to functional exhaustion. Consequently, FFA release declines, forcing the body to revert to catastrophic protein catabolism in skeletal muscle to meet energy needs (57). This shift precipitates a chronic energy crisis and a wasting state, ultimately accelerating the progression toward multiple organ failure.

In summary, adipose tissue provides energy substrates via canonical and non-canonical lipolysis, while BAT clears lipotoxicity through thermogenesis. The functional integrity of this system is central to maintaining energy homeostasis and preventing lipotoxicity during sepsis. Its dysfunction, however, accelerates the progression towards cachexia and multiple organ failure.

#### 4.1.2 Signaling regulation: the immunological buffering role of adipose tissue

##### 4.1.2.1 Inflammatory sensing by adipocytes

Contrary to previous understanding, adipocytes are far from being inert energy storage units. Instead, they are specialized inflammatory sensors equipped with various pattern recognition receptors and cytokine receptors.1. Pattern Recognition Receptor Signaling: Lipopolysaccharide (LPS) activates the MyD88/NF-κB pathway via TLR4, driving adipocytes to produce pro-inflammatory factors themselves and initiating metabolic reprogramming (69). Concurrently, mtDNA leaked from cellular stress activates the cGAS-STING pathway. The activated STING protein translocates to the MAMs, tightly linking organelle damage to the type I interferon response (45).2.Cytokine Receptor Signaling: Adipocytes directly respond to high levels of environmental cytokines such as TNF-α and IL-1β. Signaling through pathways like JAK-STAT, NF-κB, and MAPK collectively shapes their inflammatory phenotype and metabolic output, exemplifying the deep integration of immune and metabolic networks (70).3. Endoplasmic Reticulum Stress: Inflammation and metabolic disturbances trigger ER stress, activating the UPR. While a moderate UPR is protective, its sustained activation—through branches such as IRE1α and PERK—exacerbates inflammation and leads to insulin resistance (71). In summary, adipose tissue perceives inflammatory signals via pattern recognition receptors and achieves immunological buffering through dynamic shifts in its adipokine secretion profile. The integrity of this ‘sensing-regulation’ circuit is crucial for disease tolerance; its disruption leads to uncontrolled inflammation and tissue damage.

##### 4.1.2.2 Immunoregulation by adipokine

Following the perception of inflammatory signals, adipocytes execute a crucial ‘immunological buffering’ role by altering their secretory profile of adipokines.1. Early Pro-inflammatory Alarm: In the acute phase, leptin and resistin rapidly increase as pro-inflammatory alarm signals. A 2010 study by Tschöp J et al. found that leptin levels surge dramatically during early sepsis, playing a key role in enhancing early immune defense (72), Concurrently, clinical observations by Sundén-Cullberg et al. in 2007 confirmed that resistin levels are significantly elevated early in sepsis and positively correlate with disease severity (73). Elevated leptin levels promote Th1 differentiation and M1 macrophage polarization via the Ob-Rb/JAK2-STAT3 pathway, amplifying pro-inflammatory cytokine production (74). Resistin potently induces TNF-α and IL-6 and activates endothelial cells, exacerbating systemic inflammation (75).2. Mid-to-Late Phase Anti-inflammation and Protection: Adiponectin, the most important protective adipokine, exerts its effects by activating AMPK and PPARα. This leads to suppression of the NF-κB pathway, driving M2 macrophage polarization, improving insulin sensitivity, and protecting endothelial function (76). However, in severe or chronic sepsis, the adipokine network becomes dysregulated: adipose tissue dysfunction leads to ‘leptin resistance’ (77)and a relative deficiency in adiponectin levels (67). This shift towards a sustained pro-inflammatory state disrupts the immunological buffering capacity, resulting in a concurrent state of immune paralysis and persistent low-grade inflammation. This failure to effectively limit tissue damage ultimately signifies the collapse of the disease tolerance mechanism (70).

The realization of adipose tissue’s functions relies on precise regulation by immune cells. Different immune cell subtypes directly shape the dynamic characteristics of adipose metabolism through the secretion of cytokines and expression of surface molecules.

### 4.2 Precise immunomodulation of adipose metabolism: cellular subtypes and mechanisms

#### 4.2.1 B cells: the “negative regulator” of adipose lipolysis

Traditionally, the function of B cells was confined to antibody production. However, cutting-edge research has expanded their role into the realm of metabolic regulation. For instance, Gencer Sancar et al. discovered that B1a cells directly suppress adipose tissue lipolysis via the TGF-β/PDE3B axis, acting as a ‘brake’ to prevent lipotoxicity (78). Concurrently, Ippei Shimizu et al. demonstrated that regulatory B cells (Bregs) can ameliorate adipose tissue inflammation and insulin resistance (79). These findings collectively establish specific B cell subsets as crucial negative regulators in maintaining metabolic homeostasis Mechanistically, recruited or activated B cells secrete TGF-β, which acts on adipocytes to significantly upregulate the expression and activity of PDE3B (31).PDE3B subsequently hydrolyzes the intracellular second messenger cyclic AMP (cAMP), leading to decreased PKA activity. This cascade ultimately results in the inadequate phosphorylation and activation of HSL—the key rate-limiting enzyme in lipolysis—thereby potently inhibiting fat breakdown (80).This mechanism constitutes a robust negative feedback loop designed to prevent excessive and sustained lipolysis. Uncontrolled lipolysis releases an overflow of free fatty acids into the circulation, which can trigger lipotoxicity by activating pathways such as TLR4, ultimately impairing the function of the liver, pancreas, and vascular endothelium (35).

#### 4.2.2 Macrophages: the “bifunctional switch” for adipose lipolysis

As the most abundant immune cells in adipose tissue, macrophages serve as a ‘bifunctional switch’ for lipolysis due to their remarkable functional plasticity.1. Pro-lipolytic Switch (M1 Phenotype): During early sepsis, M1 macrophages are activated and infiltrate adipose tissue in large numbers, driving lipolysis to fuel the immune response. These cells produce high levels of IL-1β. Research demonstrates that IL-1β strongly promotes fat breakdown by activating the ERK signaling pathway in adipocytes, leading to direct phosphorylation and activation of HSL (15).2. Anti-lipolytic Switch (M2 Phenotype): During disease progression or the resolution phase, M2 macrophages become dominant. They create an inhibitory microenvironment through the secretion of anti-inflammatory factors such as IL-10 and TGF-β (52). Furthermore, their high expression of metabolic enzymes like arginase-1 indirectly alters the metabolic state of adipocytes by depleting local substrates (81). The function of M2 cells is not to directly inhibit lipolytic enzymes, but rather to create conditions for terminating lipolysis and initiating tissue repair by suppressing the overall inflammatory tone and remodeling the metabolic microenvironment (52, 82).

#### 4.2.3 T cells: emerging modulators of adipose-immune crosstalk

T lymphocytes, through their various subsets, are deeply involved in the immunometabolic dialogue within adipose tissue. Treg cells exert a direct inhibitory effect on lipolysis by expressing CD73, which converts local AMP into adenosine. Adenosine then binds to the A2B receptor on adipocytes, reducing intracellular cAMP levels and directly suppressing fat breakdown. Simultaneously, the IL-10 secreted by Tregs can enhance TGF-β production by B cells, indirectly reinforcing the suppression of lipolysis and forming a coordinated ‘Treg-B cell’ regulatory circuit. CD4+ T helper cells shape the global immune milieu through their signature cytokines: Th1 cells secrete IFN-γ, a classic signal for M1 macrophage polarization, thereby indirectly supporting a pro-lipolytic environment; Th2 cells, by secreting IL-4 and IL-13, drive M2 macrophage differentiation, indirectly contributing to inflammation resolution and the termination of lipolysis (50).

In contrast, cytotoxic CD8+ T cells often play a detrimental role in pathological states. During chronic inflammation, they infiltrate adipose tissue and, through the secretion of factors like IFN-γ and TNF-α, drive inflammation, recruit and activate macrophages, thereby exacerbating tissue damage and metabolic dysfunction (83).The key cellular players can be seen in the **Table 1**.

**Table 1:** Key cellular players in the adipose-immune-metabolic axis and their functions during sepsis.

Cell type	Major subtype/phenotype	Baseline function in adipose tissue	Functional remodeling during sepsis	Key metabolic pathways/molecules regulated
Adipocytea	WAT、BAT、Beige	Energy storage (WAT), thermogenesis (BAT), adipokine secretion.	Transforms from passive energy reservoir to active immunometabolic hub; enhances lipolysis to release FFAs/glycerol; secretes stage-specific adipokines (leptin, adiponectin).	HSL/ATGL (lipolysis); UCP1 (thermogenesis); TLR4/NF-κB (inflammatory sensing); Adiponectin/AMPK (anti-inflammatory).
Macrophage	M1	Pro-inflammatory, pathogen clearance.	Early infiltration, drives non-canonical lipolysis via IL-1β/ERK-HSL axis; amplifies inflammatory storm.	IL-1β→ERK→HSL; TNF-α→JNK/ERK; TLR4/FFA.
	M2	Anti-inflammatory, tissue repair.	Dominant in subacute phase; promotes inflammation resolution, tissue repair, and metabolic stability via IL-10/TGF-β.	L-10/TGF-β→STAT3/Smad; PPARγ/Arg-1.
B cell	B1, B2, Regulatory B (Breg)	Antibody production, immune regulation.	Acts as “negative regulator” of lipolysis; secretes TGF-β to upregulate PDE3B, hydrolyzes cAMP, and suppresses HSL activity; prevents lipotoxicity.	TGF-β→Smad2/3→PDE3B↑→cAMP↓→HSL inhibition.
T cell	Regulatory T (Treg)	Immune suppression, metabolic homeostasis.	Suppresses lipolysis via CD73-adenosine-A2BR axis (reduces cAMP); enhances TGF-β production; promotes M2 polarization.	CD73→Adenosine→A2BR→AC inhibition→cAMP↓.
	Th1	Cell-mediated immunity.	Secretes IFN-γ, promotes M1 polarization, indirectly supports pro-inflammatory and lipolytic milieu.	IFN-γ→JAK/STAT1→M1 polarization.
	Th2	Humoral immunity, anti-parasite.	Secretes IL-4/IL-13, drives M2 polarization, supports inflammation resolution.	IL-4/IL-13→STAT6→M2 polarization.
	Cytotoxic CD8+	Cytotoxicity, viral clearance	Infiltrates in chronic phase; secretes IFN-γ/TNF-α, exacerbates inflammation and insulin resistance.	IFN-γ/TNF-α→inflammatory amplification→metabolic dysfunction.
Neutrophil	Activated (N1)	Phagocytosis, NETosis, acute defense.	Early infiltration, releases proteases/ROS, may exacerbate tissue damage and amplify inflammatory signals.	NETs→cGAS-STING; ROS→NF-κB/NLRP3.
Eosinophil	Conventional	Anti-parasitic, supports adipose homeostasis.	Numbers may decline; loss may impair M2/Treg support, worsening metabolic inflammation.	IL-5/IL-13→M2/Treg maintenance.

### 4.3 Key molecular pathways of the adipo-immune-metabolic axis: crosstalk regulation and disease tolerance

The precise regulation of the adipose-immune-metabolic axis hinges on the integration of several core signaling pathways. These pathways do not operate in isolation but engage in intricate crosstalk, collectively determining the balance between adaptive energy mobilization and maladaptive tissue damage. Below, we dissect three pivotal axes: the Insulin-INSR-Thermogenesis axis, which links nutrient sensing to thermogenic capacity; the TGFβ-PDE3b-cAMP axis, a key immune-mediated brake on lipolysis; and the STING-ER Stress-mtROS axis, a sensor of cellular damage that can drive both defense and pathology.

#### 4.3.1 The insulin-INSR-thermogenesis axis: a disease tolerance pathway transcending conventional glycemic control

During sepsis, a critical ‘Insulin-INSR-Thermogenesis Axis’ is activated. This axis exhibits synergy with the TGFβ-PDE3b-cAMP pathway: insulin, via the PI3K-Akt pathway, can inhibit Smad2/3 phosphorylation, indirectly attenuating the TGFβ-mediated upregulation of PDE3b and preventing excessive suppression of lipolysis. Conversely, overactivation of the STING-ER stress-mtROS axis can damage the insulin receptor (INSR), leading to impaired BAT thermogenic function and creating a vicious cycle of ‘pathway imbalance → functional deterioration’. Contrary to its traditional role as an anabolic hormone, insulin’s signaling output in BAT is ‘reprogrammed’ towards energy consumption (84).

**Figure 1:**
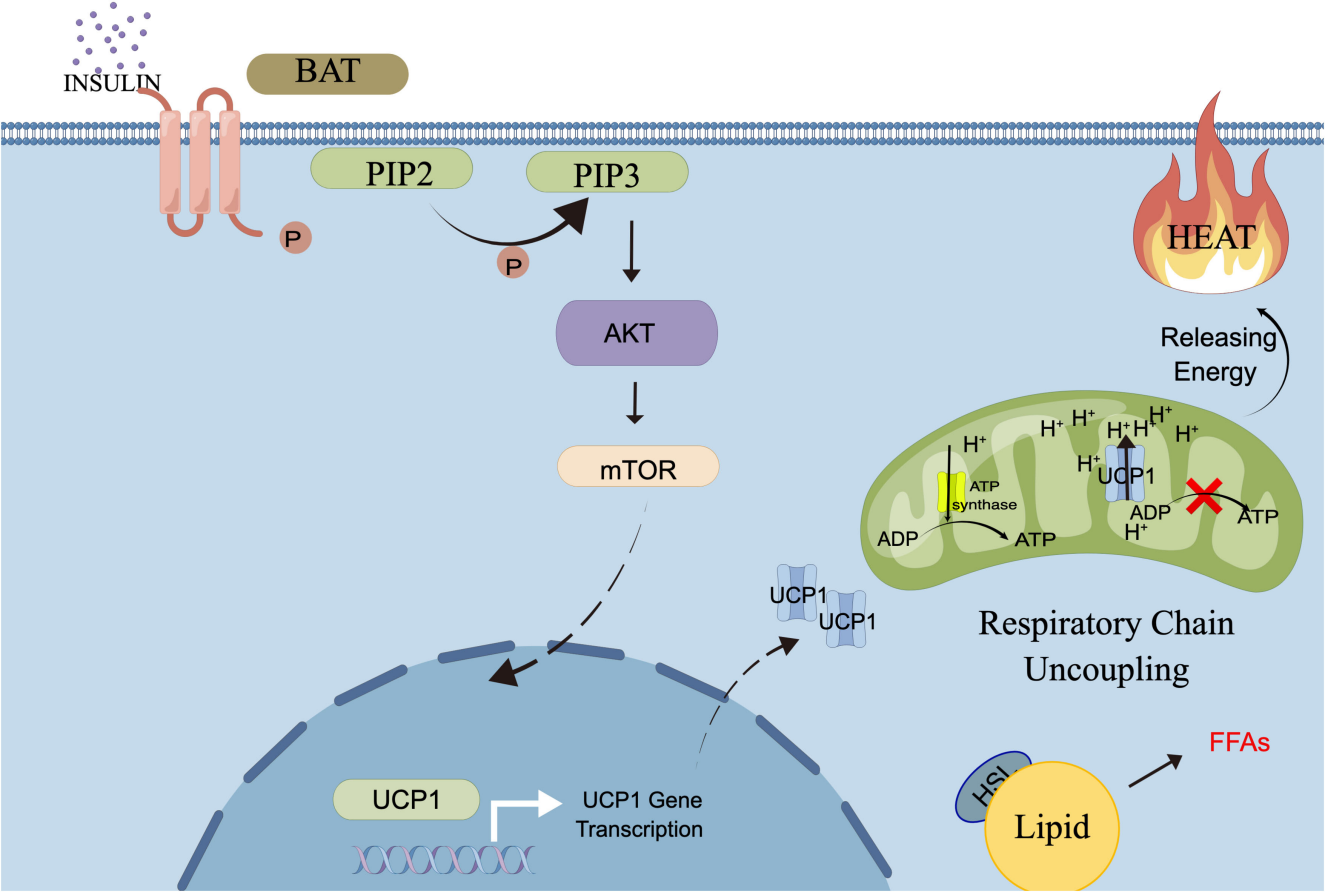
The insulin-INSR-thermogenesis axis: Schematic of the proposed insulin signaling pathway promoting thermogenesis in BAT. PIP2,Phosphatidylinositol 4; 5-bisphosphate PIP3, Phosphatidylinositol 3; 4,5-trisphosphate AKT; Protein kinase B UCP1, Uncoupling protein 1; ATP, Adenosine triphosphate ; ADP, Adenosine diphosphate; BAT, Brown adipose tissue; FFA, Free fatty acids; mTOR, mechanistic target of rapamycin; Pathway Description: Upon binding to its receptor on BAT, insulin activates the IRS1-PI3K-AKT pathway. Activated AKT promotes transcriptional upregulation of UCP1 and mitochondrial biogenesis. Increased UCP1 protein integrates into the mitochondrial inner membrane, where it uncouples the proton gradient from ATP synthesis, thereby dissipating energy derived from FFA oxidation as heat.

The core mechanisms of this axis are as follows:1. Signal Initiation and Program Execution: Upon binding to the insulin receptor on the BAT cell membrane, insulin activates the canonical IRS1-PI3K-Akt pathway. Activated Akt can directly act on the Ucp1 gene promoter, significantly upregulating its transcription and protein expression (85). Concurrently, insulin signaling synergistically promotes mitochondrial biogenesis (61), establishing the platform for thermogenesis.2. Energy Dissipation: The highly expressed UCP1 protein uncouples the mitochondrial respiratory chain, directly converting the chemical energy from substrate oxidation into heat (10). The specific process can be seen in the **Figure 1**.

Research confirms that insulin is necessary for activating BAT energy expenditure under conditions requiring thermogenesis. Therefore, in the context of sepsis, this axis consumes energy to maintain core body temperature and dissipates excess lipid energy.

The Insulin-INSR-Thermogenesis Axis does not operate in isolation; it forms an exquisite ‘Supply-Consumption’ synergy with systemic fat mobilization (lipolysis), collectively consolidating disease tolerance.1. Functional Coupling: Directed Energy Flow: The substantial amount of free fatty acids released into the circulation via lipolysis from white adipose tissue are efficiently taken up and cleared by brown adipose tissue as the primary ‘client’ (61). The actual oxidation of FFAs and uncoupling via UCP1 in BAT is primarily and directly driven by sympathetic nervous system activation (e.g., via β3-adrenergic receptors) (10).Insulin signaling, while classically anabolic in promoting lipid storage and glucose uptake in BAT (84), plays a critical indirect and supportive role in thermogenesis. Specifically, through pathways such as Akt, insulin upregulates UCP1 expression (85) and promotes mitochondrial biogenesis (86). This ‘primes’ BAT by replenishing intracellular substrate stores and enhancing its cellular capacity, thereby enabling a robust and efficient thermogenic response when catecholamine stimulation occurs. This creates an efficient metabolic pipeline: ‘WAT → circulating FFAs → BAT (insulin-activated → UCP1 thermogenesis)’, ensuring energy is rapidly and directionally utilized for survival-critical tasks.2. Homeostatic Synergy: Avoiding Metabolic Conflict: Although insulin inhibits WAT lipolysis at the systemic level (34), its thermogenic role in BAT exhibits tissue-specific differences. This synergy and dissociation enable precise regulation and systemic protection. Precise regulation is manifested by permitting moderate systemic fat mobilization while ensuring energy is diverted to BAT for ‘safe combustion’. Systemic protection is achieved by consuming excess, lipotoxic FFAs through thermogenesis, thereby protecting organs like the liver and pancreas, while the heat production itself maintains the core body temperature essential for survival.

Recent research on the Insulin-INSR-Thermogenesis Axis indicates: UCP1, as the core molecule of BAT thermogenesis, Igor Golic’s research showed that insulin can modulate mitochondrial function in brown adipose tissue, altering UCP1 expression and cellular thermogenic capacity (86). However, directly targeting UCP1 is highly challenging. Consequently, current research often focuses on its upstream pathways; for instance, Gómez-García found that CL316,243 significantly increased UCP1 protein expression in both BAT and white adipose tissue in mouse studies (87). To date, no safe and highly specific direct UCP1 agonist for humans has been identified. Agonists/inhibitors targeting upstream pathways (e.g., mirabegron derivatives) have been shown to activate BAT and improve metabolism in human clinical studies, but their application, particularly in obesity treatment, is limited by cardiovascular side effects such as increased blood pressure and heart rate (88).

#### 4.3.2 The TGFβ-PDE3b-cAMP axis: a core negative feedback pathway for metabolic homeostasis

The TGFβ-PDE3b-cAMP axis represents a classic negative feedback pathway initiated by immune cells to maintain metabolic homeostasis. Through the molecular sequence of upregulating PDE3b → degrading cAMP → inhibiting HSL/ATGL activity, it provides an essential “braking” mechanism for the excessive fat mobilization seen in sepsis. This axis is one of the key balancing acts the body relies on to achieve disease tolerance, walking a tightrope between energy mobilization and tissue protection.

**Figure 2:**
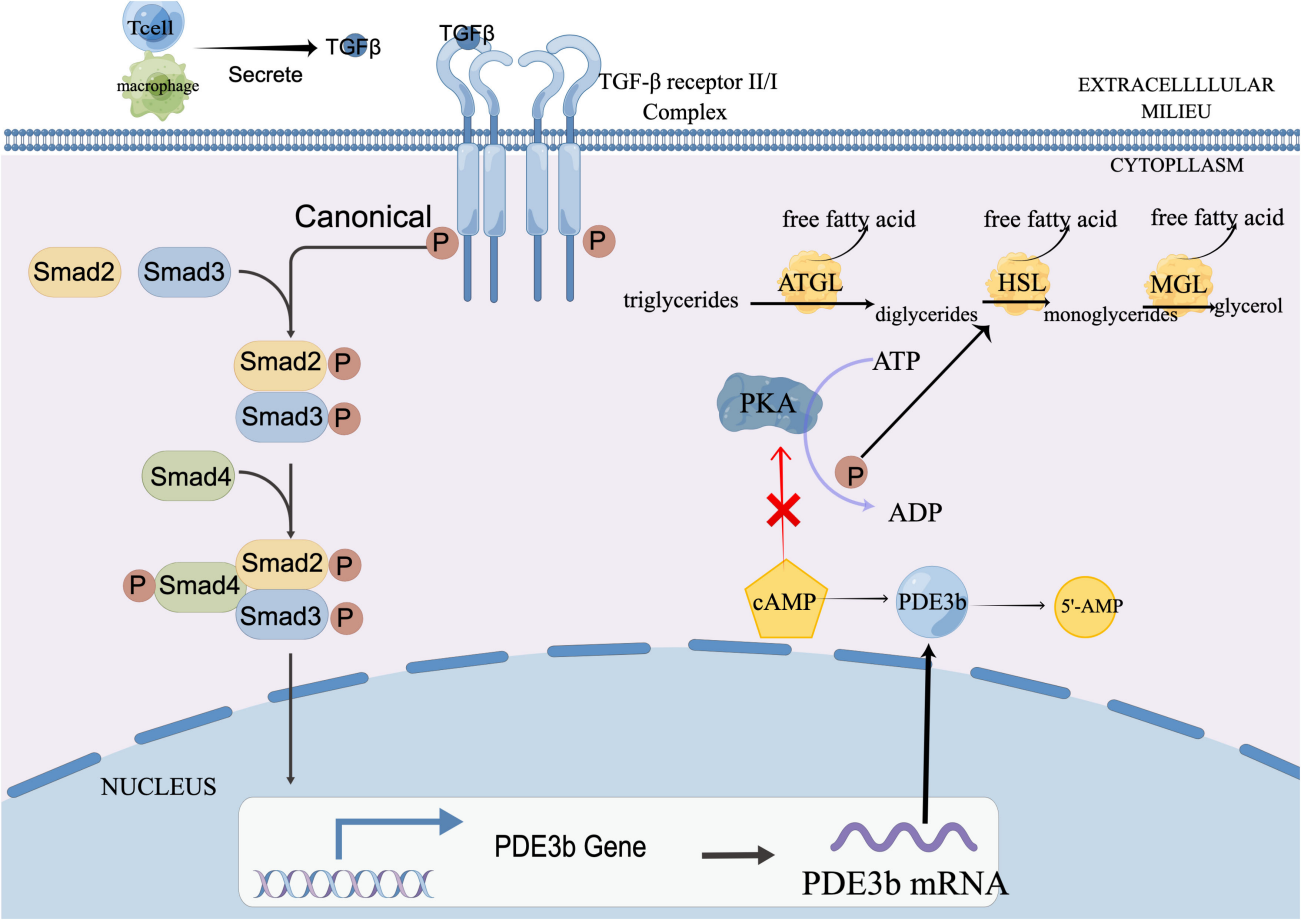
The TGFβ-PDE3b-cAMP axis: Stepwise mechanism of TGF-β-mediated suppression of lipolysis in adipocytes. ATGL, Adipose Triglyceride Lipase; cAMP, Cyclic adenosine monophosphate; HSL, Hormone-Sensitive Lipase; PDE3b, Phosphodiesterase 3B; PKA, Protein Kinase A; TGF-β, Transforming Growth Factor-beta Pathway Description:1.Signal Production: Regulatory B cells secrete the cytokine TGF-β. 2.Signal Reception & Transduction: TGF-β binds to its type II/I receptor complex on the adipocyte membrane, activating the canonical Smad (Smad2/3) transcription pathway.3. Transcriptional Regulation: The activated Smad complex translocates to the nucleus and binds to the promoter of the *PDE3b* gene, upregulating its expression.4. Activated UCP1 creates a proton leak, allowing protons (H+) to bypass ATP synthase and re-enter the mitochondrial matrix. This dissipates the proton gradient, uncoupling respiration from ATP production and releasing the energy directly as heat.

Core Mechanisms:1. Initiation of Immune Signal: Specific immune cell subsets, primarily regulatory B cells, are recruited or activated and secrete Transforming Growth Factor-β as the initiating signal.2. Receptor Binding and Signal Transduction: TGF-β binds to the TGF-β receptor II/I complex on the adipocyte membrane, activating its intrinsic serine/threonine kinase activity. This triggers the canonical Smad signaling pathway, leading to the phosphorylation of Smad2/3 and initiating downstream transcriptional reprogramming (89).3. Upregulation of the Key Effector Molecule: A central transcriptional target of TGF-β signaling is the gene for Phosphodiesterase 3B. The activated Smad complex directly binds to the PDE3b promoter, significantly upregulating its expression and activity. 4.Degradation of the Second Messenger and Halt of the Lipolytic Engine: PDE3b specifically hydrolyzes cyclic AMP, converting it to inactive 5’-AMP (34). The sharp decline in cAMP levels directly reduces Protein Kinase A activity. Inactive PKA cannot adequately phosphorylate and activate the two key rate-limiting enzymes of lipolysis—Hormone-Sensitive Lipase and Adipose Triglyceride Lipase—ultimately leading to significant suppression of fat breakdown. The specific process can be seen in the **Figure 2**.

Recent studies on the TGFβ-PDE3b-cAMP axis confirm that upregulating PDE3b expression or activity effectively inhibits lipolysis (90).Conversely, using PDE3 inhibitors stimulates lipolysis and increases plasma free fatty acid levels (91)These findings suggest that the activation of this axis holds pathophysiological significance far beyond simple signal transduction; it constitutes a core defensive strategy for maintaining homeostasis during the metabolic storm of sepsis. Firstly, as a “precision brake,” this pathway effectively prevents runaway lipolysis driven by pro-inflammatory signals and the premature depletion of energy reserves. Secondly, avoiding lipotoxicity is its most critical protective role. Uncontrolled lipolysis releases excessive FFAs into the circulation, leading to their ectopic deposition in non-adipose organs like the liver and pancreas, potentially causing mitochondrial dysfunction, insulin resistance, and even cell apoptosis. The essence of this axis is “Bidirectional Immunometabolic Crosstalk.” It exemplifies the immune system’s capacity not only to drive inflammation and metabolism but also to proactively downregulate these processes, showcasing a sophisticated layer of metabolic regulation vital for host defense.

#### 4.3.3 The STING-ER stress-mtROS axis: a vicious cycle driving inflammation and cell death in sepsis

**Figure 3:**
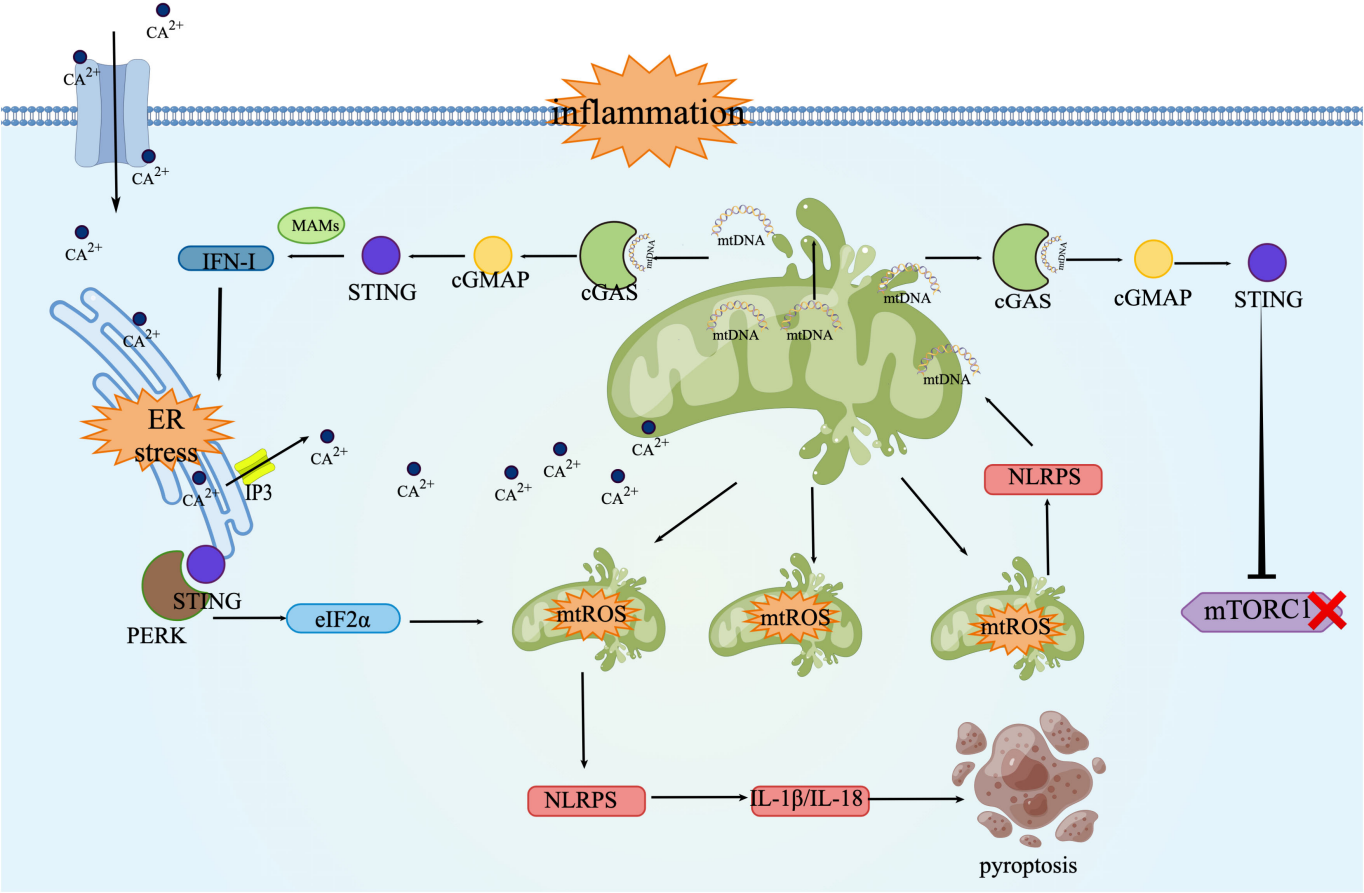
The STING-ER Stress-mtROS axis: The STING-ER Stress-mtROS axis forms a vicious cycle driving inflammation and cell death in sepsis. cGAS, Cyclic GMP-AMP synthase; ER, Endoplasmic reticulum; eIF2α, Eukaryotic initiation factor 2 alpha; IFN-I, Type I interferon; IL-1β/IL-18, Interleukin-1 beta/Interleukin-18; IP3, Inositol trisphosphate; MAMs, Mitochondria-associated; ER, membranes; mtDNA, Mitochondrial DNA; mtROS, Mitochondrial reactive oxygen species; NLRP3, NOD-, LRR- and pyrin domain-containing protein 3; PERK, Protein kinase RNA-like ER kinase; STING, Stimulator of interferon genes; UPR, Unfolded protein response Pathway Description. This schematic illustrates a proposed intracellular vicious cycle central to septic pathology. Initial insults lead to mitochondrial damage and cytosolic release of mtDNA. mtDNA is sensed by cGAS, which produces cGAMP to activate STING. Activated STING triggers IFN-I production and, by leaving the ER, induces ER stress and the UPR (e.g., PERK-eIF2α branch). ER stress promotes Ca²^+^ release via channels like IP3R. Through MAMs, this calcium floods into mitochondria, causing calcium overload, electron transport chain disruption, and a burst of mtROS. STING also suppresses mTORC1, potentially impairing cellular stress responses. mtROS further damages mitochondria, oxidizing mtDNA to enhance its immunogenicity, thereby creating a positive feedback loop that reactivates cGAS-STING. Simultaneously, mtROS serves as a key signal for NLRP3 inflammasome assembly, leading to the maturation of IL-1β/IL-18 and induction of inflammatory pyroptosis.

In sepsis, the ‘STING-ER Stress-mtROS Axis’ forms the core of a vicious cycle that drives persistent inflammation and cellular apoptosis. Initial inflammation and hypoxia damage mitochondria, leading to the leakage of mtDNA into the cytosol (45), Cytosolic mtDNA is recognized by cGAS, which generates cGAMP to activate the STING signal (92). Activated STING not only translocates to MAMs to drive type I interferon expression, but its depletion from the endoplasmic reticulum also induces ER stress (93), This activates pathways like the UPR’s PERK-eIF2α branch, which, while attempting to restore homeostasis, paradoxically exacerbates inflammatory signaling (94). ER stress causes the release of calcium ions through channels like IP3R, which are then taken up in large quantities by adjacent mitochondria via MAMs. Mitochondrial calcium overload disrupts the electron transport chain, causing electron leakage and a burst in superoxide anion production, known as mtROS burst (95). Furthermore, activated STING can suppress mTORC1 activity, and this inhibition can alter the cell’s antioxidant defense capacity, potentially indirectly worsening oxidative stress (96).mtROS further exacerbates mitochondrial damage by oxidizing mtDNA itself, making it more recognizable to cGAS or more potent at activating NLRP3. On one hand, this creates a positive feedback loop, releasing more mtDNA to re-activate the cGAS-STING pathway (97). On the other hand, mtROS acts as a potent activation signal, directly triggering the assembly of the NLRP3 inflammasome, promoting the maturation of IL-1β/IL-18 and inducing pyroptosis (44). The specific process can be seen in the **Figure 3**.

The role of the STING-ER stress-mtROS axis in sepsis exhibits a distinct stage-dependent duality Early Phase: Moderate activation of the axis plays an adaptive defense role. Appropriate type I interferon production is crucial for coordinating early anti-infective immunity (98), while the initiated UPR aims to restore ER homeostasis and promote cell survival (94, 98).At this stage, inflammation and oxidative stress are controlled, protective responses designed to eliminate the threat. Advanced Phase: When sepsis becomes uncontrolled and progresses to a later stage, the axis descends into a self-sustaining vicious cycle. Persistent type I interferon response paradoxically promotes immune paralysis and tissue damage (96), Severe and irreversible ER stress switches its signaling from “pro-survival” to “pro-apoptosis” by activating pathways involving CHOP (99), Concurrently, explosive mtROS strongly activates the NLRP3 inflammasome, driving pyroptosis and leading to widespread death of adipocytes, immune cells, and parenchymal cells (44).On a metabolic level, mitochondrial failure directly impairs ATP synthesis (100), and disrupts normal lipid metabolism, exacerbating lipolytic failure (70). Ultimately, these cellular catastrophes converge into multiple organ dysfunction (101).

Recent single-cell multi-omics studies have revealed that exosomes secreted by adipocytes during the chronic phase of sepsis can carry STING and trigger a potent type I interferon response via the cGAS-STING pathway, persistently amplifying inflammation (102, 103)Conversely, using specific small-molecule STING inhibitors (e.g., H-151) can effectively block this signaling axis, significantly attenuating tissue damage and improving outcomes in inflammatory and sepsis models (104). This collectively confirms that targeting STING-mediated communication between adipose and immune cells represents a potential therapeutic strategy.

The three key pathways described above—the Insulin-INSR-Thermogenesis Axis, the TGFβ-PDE3b-cAMP Axis, and the STING-ER Stress-mtROS Axis—orchestrate the function of the adipo-immune-metabolic axis through intricate crosstalk. A balance among these pathways enhances disease tolerance, whereas their imbalance leads to organ failure. Their stage-dependent nature provides molecular targets for precise intervention.

In summary, the Insulin-INSR axis supports energy expenditure and substrate storage, the TGFβ-PDE3b axis provides critical feedback inhibition to prevent lipotoxicity, and the STING-mtROS axis integrates organelle stress with immune activation. Their balanced interaction facilitates disease tolerance (e.g., coordinated fuel supply and inflammation buffering in the subacute phase), whereas their dysregulation (e.g., sustained STING activation coupled with insulin resistance) propels the transition to metabolic exhaustion and organ failure. The stage-specific activity of these pathways offers a molecular basis for the ‘Stage-Specific Adaptation’ hypothesis.

### 4.4 Dynamic characteristics of the adipo-immune-metabolic axis across sepsis stages

#### 4.4.1 Acute phase (Early, ~0–48 hours): “inflammatory storm and energy crisis”

This stage is dominated by the pro-lipolytic arm of ‘Bidirectional Immunometabolic Crosstalk’, embodying the early rapid response mode of the ‘Stage-Specific Adaptation’ hypothesis. Its core feature is the host’s initiation of the fastest defense mechanisms to counter the sudden life-threatening insult. Although fat mobilization is initiated, the drastic breakdown of skeletal muscle protein via the ubiquitin-proteasome pathway becomes dominant (20), providing the most rapid energy substrates for the brain and immune cells. Immunologically, a pro-inflammatory response holds absolute sway, with neutrophils and M1 macrophages infiltrating adipose tissue and releasing high levels of TNF-α, IL-1β, and IL-6 (105), They directly ‘accelerate’ energy mobilization via the IL-1β→ERK-HSL pathway (15).The metabolic-immune profile of this phase represents an adaptive program designed for a rapid response to a life threat (18), Consequently, clinical management focuses primarily on fluid resuscitation, source control, and antibiotic administration (106), rather than sophisticated metabolic support.

#### 4.4.2 Subacute phase (Intermediate, 3–7days): “metabolic defense and immunoregulation”

This stage is the core manifestation of the ‘Metabolic Defense Priority’ hypothesis, where fat mobilization and protein sparing peak, and bidirectional immunometabolic crosstalk achieves a precise balance. Once the acute threat is initially controlled, the body switches to an ‘endurance race’ mode, and the function of the adipo-immune-metabolic axis peaks, exhibiting refined regulation. Metabolically, lipolysis reaches its zenith and becomes the central energy source, coupled with significantly enhanced hepatic ketogenesis (17), Together, they execute the ‘Metabolic Defense Priority’ strategy, aiming to maximize protein sparing and protect muscle function (18). Concurrently, brown adipose tissue is activated, consuming FFAs via UCP1-mediated thermogenesis to maintain body temperature. Immunologically, the landscape undergoes profound remodeling, shifting towards M2 macrophages, Tregs, and Bregs, with rising levels of anti-inflammatory cytokines like IL-10 and TGF-β (17), This exemplifies precise ‘Bidirectional Immunometabolic Crosstalk’: while M1/IL-1β signaling persists, the Breg/TGF-β → PDE3b-cAMP axis is activated as a crucial ‘brake’ to prevent runaway lipolysis. Therefore, this phase represents the ‘golden window’ for clinical nutritional support and metabolic intervention, requiring the provision of nutrients suitable for fat oxidation and aggressive protein supplementation to prevent excessive muscle breakdown (107, 108).

#### 4.4.3 Chronic phase/PICS phase (Late, >7 days): “metabolic exhaustion and immune dysregulation”

**Figure 4:**
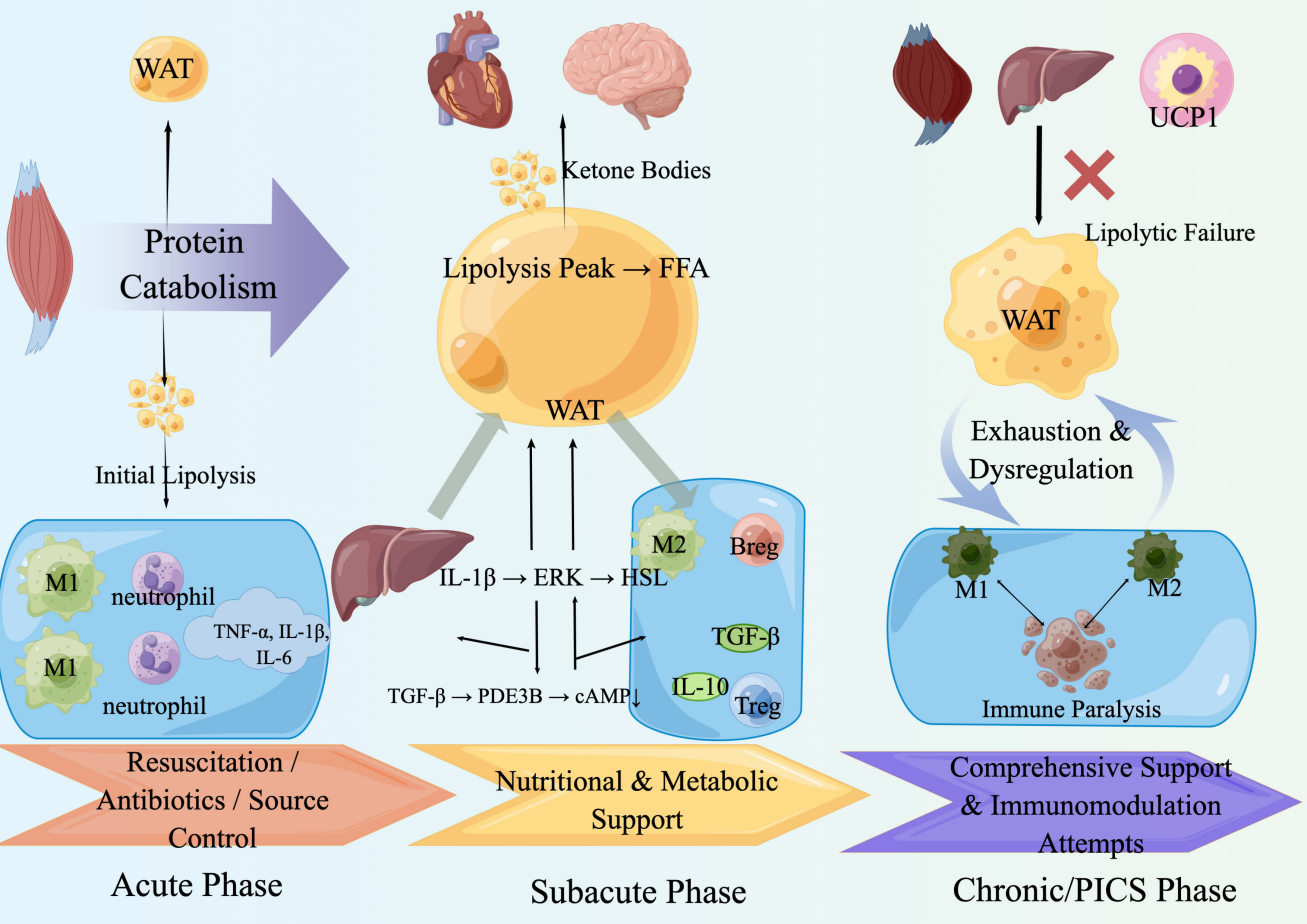
Stage-specific reprogramming of the adipose-immune-metabolic axis during sepsis progression. ERK, Extracellular signal-regulated kinase; FFA, Free fatty acids; HSL, Hormone-sensitive lipase; IL-1β, IL-6, IL-10, TNF-α, Interleukin-1 beta, -6, -10, Tumor necrosis factor-alpha; PICS, Persistent inflammation, immunosuppression, and catabolism syndrome; TGF-β, Transforming growth factor-beta; UCP1, Uncoupling protein 1; WAT, White adipose tissue; Annotation Acute Phase: Dominated by a catabolic surge in WAT, resulting in a lipolysis peak and systemic inflammation driven by M1 macrophages/neutrophils. Subacute Phase: Characterized by immune phenotype switching, with the rise of M2 macrophages and regulatory cells secreting anti-inflammatory mediators (e.g., IL-10, TGF-β), alongside attempts at comprehensive metabolic and immune support. Chronic/PICS Phase: Marked by systemic exhaustion & dysregulation, featuring lipolytic failure, persistent protein catabolism, and a state of immune paralysis.

The transition mechanisms outlined in the ‘Stage-Specific Adaptation’ hypothesis fail, and the adipo-immune-metabolic axis collapses into a vicious cycle of ‘metabolic exhaustion - immune paralysis’. If sepsis persists unresolved, the body enters a state of exhaustion, and axis function breaks down. Metabolically, adipose tissue exhibits severe lipolytic failure due to insulin resistance and direct inflammatory suppression (109, 110).Accompanied by hypertriglyceridemia (111).Widespread mitochondrial dysfunction leads to disordered energy metabolism, forcing the body back to a reliance on the catastrophic breakdown of skeletal muscle protein, ultimately resulting in severe cachexia and multiple organ failure (20). Immunologically, this stage is characterized by immune paralysis, featuring lymphocyte apoptosis, exhaustion, and anergy (4)Treatment at this stage is extremely challenging and necessitates aggressive exogenous intervention, including meticulous metabolic (e.g., insulin therapy), targeted nutritional support (e.g., specific lipid supplementation and adequate protein), and attempted immunomodulation, aiming to break this lethal vicious cycle (107, 112). The specific process can be seen in the **Figure 4**.

## 5 Challenges and controversies: limitations and unresolved issues in current research

### 5.1 Challenges at the mechanistic level: unclear precision regulation of cellular subtypes and metabolites

Although current research has identified numerous cellular subtypes within adipose tissue and the immune system, significant knowledge gaps remain regarding their precise identities, dynamic changes, and specific functions across different stages of sepsis. The role of ketone bodies in sepsis extends far beyond their classical perception as alternative fuel sources; however, whether their overall effect is protective or detrimental remains unresolved and contentious. Challenges at the mechanistic level are not only reflected in the unclear precise regulation of cells and metabolites but also involve missing details in the regulation of the core pathways discussed previously (e.g., the STING-ER Stress-mtROS Axis, the MAMs-STING Hub). Key unanswered questions include: Do differences exist in the activation threshold of STING between adipocytes and immune cells? How does the MAMs-mediated Ca²^+^ flux precisely coordinate the regulation of adipokine secretion and immune cell polarization?

#### 5.1.1 Insufficient subcategorization of adipose-immune interacting cell subtypes

Despite our growing understanding of the adipo-immune-metabolic axis in sepsis, significant blind spots remain in the current knowledge of its key cellular subtypes. In the B-cell domain, the dynamic changes of subsets like B1, B2, and B10 across different septic stages and their specific roles in regulating lipolysis are not clearly defined; The functional differences between tissue-resident macrophages (e.g., Lipid-Associated Macrophages) and monocyte-derived infiltrating macrophages in sepsis have not been distinguished. The pathological significance of their spatial distribution (e.g., whether they form “crown-like structures”) still requires validation (113).The impact of adipocyte depot specificity (subcutaneous vs. visceral) and preadipocyte differentiation fate on long-term sepsis outcomes currently lacks direct evidence. Furthermore, the traditional M1/M2 dichotomy is inadequate to cover the full functional heterogeneity of macrophages. The roles of novel macrophage subtypes revealed by single-cell sequencing (e.g., lipid-associated macrophages or other metabolically regulatory states) in sepsis have not been sufficiently elucidated (113).The spatiotemporal dynamics—specifically, the spatial distribution (e.g., forming “crown-like structures” around dead adipocytes vs. diffuse infiltration) and temporal evolution of different macrophage subtypes/states across sepsis stages (114) require more research. Most critically, how sepsis influences the differentiation fate of adipose precursor cells, thereby determining the long-term plasticity and function of adipose tissue (115), remains largely unknown.

#### 5.1.2 Controversies regarding the specific mechanisms of metabolite action

Current research confirms that the functions of key metabolites are highly context-dependent, yet a complete framework to guide precise intervention is still lacking. For instance, the groundbreaking study by Youm et al. demonstrated that the ketone body β-hydroxybutyrate can directly inhibit the NLRP3 inflammasome by acting as an HDAC inhibitor, suggesting ketone bodies play a crucial role as inflammatory ‘brake’ signals beyond their classical function as an alternative fuel (24). However, this protection is not universal. Research by Wang et al. further revealed that a ketogenic state, which is protective in bacterial infections, might be detrimental in viral infections (18). These findings highlight that ketone bodies have transcended their classic role to become important signaling molecules. While they can exert potent organ protection via HDAC inhibition and NLRP3 blockade ( (24), extremely high blood ketone levels are also a marker of poor prognosis, indicating a narrow ‘therapeutic window’ (100). The type of fatty acid dictates its biological effect: Saturated fatty acids can drive inflammation and lipotoxicity via pathways such as TLR4 activation (35). Among unsaturated fats, Omega-6 fatty acids (e.g., arachidonic acid) are primary precursors for potent pro-inflammatory eicosanoids (e.g., prostaglandins, leukotrienes). In contrast, Omega-3 fatty acids (e.g., EPA, DHA) actively promote inflammation resolution by serving as substrates for pro-resolving mediators (e.g., Resolvins, Protectins) and by activating anti-inflammatory pathways such as those mediated by PPARs (116).The role of adenosine is most contentious: locally and early, it exerts powerful anti-inflammatory and organ-protective effects via the A2A receptor (117), However, at the systemic level and in later stages, sustained adenosine signaling becomes a key mediator driving immune paralysis and secondary infections (4), making antagonism of its signaling (e.g., using A2A receptor antagonists) a potential strategy to restore immune function.

The lack of clarity regarding precise mechanistic regulation is further amplified by the disparities between animal models and human sepsis, hindering the translation of basic research findings to the clinical setting.

### 5.2 Limitations at the model level: disparities between animal models and human sepsis

Current animal models of sepsis, such as Cecal Ligation and Puncture (CLP) and Lipopolysaccharide (LPS) injection, provide controlled, reductionist systems that mimic specific aspects of the human condition. These models are indispensable for mechanistic discovery but inherently simplify the extreme etiological and clinical heterogeneity of human sepsis, which stems from diverse infection sources, pathogens, and vastly heterogeneous patient populations (in terms of age, comorbidities, and genetics).

#### 5.2.1 Species differences leading to challenges in extrapolation

Differences in Metabolic Pathways:1.BAT Disparity: Adult mice possess substantial amounts of highly active brown adipose tissue, which is a core organ for maintaining body temperature in response to cold and environmental challenges. In contrast, the amount and activity of BAT in healthy adult humans are significantly lower than in mice and decline with age and obesity. Human thermoregulation relies more on skeletal muscle, vasoconstriction, and potentially beige adipose tissue. Consequently, the universality and importance of BAT-driven protective thermogenesis pathways, discovered in murine models, may be overestimated for human sepsis. 2. Basal Metabolic Rate and Response Dynamics: Mice have an extremely high metabolic rate, leading to more intense and rapid metabolic responses to starvation and stress. Humans, however, have a relatively lower metabolic rate and a more protracted disease course. Therefore, the rapid and drastic metabolic switches observed in mice may occur more slowly and gradually in humans. Disease stage definitions based on murine timelines may not accurately correspond to human disease progression. Furthermore, the reliance on and regulation of alternative fuels like ketone bodies likely differ between species.3. Lipoprotein Profiles: The lipoprotein profile of mice differs from that of humans. Consequently, mechanisms underlying hypertriglyceridemia and adipose tissue lipolytic failure identified in mouse models may not fully reflect the reality of lipid disorders in human sepsis.

Differences in Infection Models:1. Limitations of Standard Models: Although the mouse CLP model is considered the ‘gold standard’, it still represents a standardized, focal peritoneal infection. The LPS model provides only a single, potent inflammatory stimulus and does not recapitulate the natural process of bacterial proliferation and clearance. Therefore, mechanisms of adipo-immune-metabolic regulation discovered in a single model might only be applicable to a specific subset of human sepsis (e.g., abdominal origin for CLP) and cannot be generalized to sepsis stemming from other infection sources.2. Lack of Clinical Comorbidities: Experimental mouse models typically use young, genetically identical animals without comorbidities. Human patients, however, often present with multiple underlying conditions. A strategy proven to improve survival by enhancing lipolysis in healthy young mice might trigger catastrophic lipotoxicity and potentially accelerate mortality in septic patients with pre-existing conditions like diabetes and dyslipidemia.

#### 5.2.2 Research gap: sex and age factors

In studies investigating the adipo-immune-metabolic axis in sepsis, the vast majority of foundational experiments conventionally use young male mice by default, aiming to circumvent variability associated with the female estrous cycle. However, this practice creates a significant disconnect from clinical reality and risks developing therapeutic strategies that are ineffective or even harmful for specific demographic groups, such as women and the elderly.

1. Sex-Specific Differences: Sex hormones play a crucial role. Estrogen may confer protection mediated by Estrogen Receptor α (ERα). Specifically, 17β-estradiol possesses immunomodulatory and vasoprotective properties and may enhance both innate and adaptive immunity via ERα signaling (118, 119), On the other hand, the influence of the sex chromosome complement is often entirely overlooked. Females, due to the X-chromosome immune gene dosage effect, may possess a more robust and diverse immune response capability (120).2. The Age Dimension: The standard use of young models fails to recapitulate key features of the aged host. This includes immunosenescence—characterized by the coexistence of inflammaging and T cell exhaustion (112) and metabolic aging, featuring dysfunctional adipose tissue and attenuated mitochondrial function (121).

These model limitations constrain the depth of mechanistic investigation, and the resultant insufficient mechanistic understanding directly hinders clinical translation, thereby creating a vicious ‘research-translation gap’.

### 5.3 Challenges and obstacles in clinical translation

The blind spots in mechanistic research and the limitations of model systems directly lead to two core, interconnected obstacles in clinical translation: the difficulty in deciphering patient heterogeneity and the challenge of developing viable therapeutic targets. Unclear mechanisms result in a lack of biomarkers, while model biases lead to failed target validation.

#### 5.3.1 Patient heterogeneity and the predicament of precision medicine

Sepsis is not a single disease but a highly heterogeneous syndrome driven by different infection sources, pathogens, host genetic backgrounds, and comorbid states (2, 122).This heterogeneity is the primary obstacle to clinical translation.1. The Dynamic Disease Course vs. Timing of Intervention: The progression of sepsis is dynamic, and the function of the adipo-immune-metabolic axis evolves from compensation to decompensation. According to the ‘Stage-Specific Adaptation’ hypothesis, patients have distinct characteristics in the acute, subacute, and chronic phases. However, it is clinically challenging to precisely determine a patient’s current stage, making correctly timed interventions difficult.2. The Double-Edged Sword Effect of Metabolic Interventions and Safety Concerns: Targeting metabolic pathways is attractive but fraught with risk, as metabolites themselves are pleiotropic and concentration-dependent. Supplementing ketone bodies may be protective early on by inhibiting HDAC and the NLRP3 inflammasome, but in later stages, it could exacerbate immune paralysis and acidosis. Driving lipolysis can supply energy, but excessive free fatty acids can induce lipotoxicity, impairing liver and pancreatic function. Adenosine has anti-inflammatory effects early but contributes to immune paralysis in later stages.

#### 5.3.2 Lack of biomarkers and therapeutic targets

Currently, no single or panel of metabolites/adipokines has been validated for bedside use in stratifying, staging, or predicting the prognosis of septic patients. Many potential biomarkers, such as FABP5 identified by Rong Wu et al. as having a pro-inflammatory role in sepsis, remain in the animal model stage, far from clinical verification and application (123), Many molecular targets effective in animal models (e.g., STING, specific B-cell subsets) lack safe and effective agonists or antagonists for human use. Directly modulating these pathways may lead to unforeseen off-target effects. Intervention tools are also severely lacking. Currently, there are no safe, specific UCP1 agonists available for human use (124). Non-specific drugs (e.g., β3-adrenergic receptor agonists) can cause serious cardiovascular side effects, such as tachycardia and hypertension (125, 126).

### 5.4 Clinical challenges: the dual role of obesity in sepsis

The chronic low-grade inflammation (“metaflammation”) present in obese individuals, characterized by adipose tissue macrophage infiltration and a pro-inflammatory adipokine profile, establishes a pre-activated host state that complicates sepsis management (67). This presents two major, interrelated clinical challenges: the variable impact on sepsis pathophysiology and the paradoxical clinical outcomes observed.

#### 5.4.1 Pathophysiological amplification vs. metabolic reserve

Pre-existing adipose tissue inflammation and insulin resistance may predispose patients to a more severe septic course. The diminished anti-inflammatory buffering capacity can exacerbate the early cytokine storm, while impaired metabolic flexibility may disrupt the protective “Metabolic Defense Priority” response, increasing the risk of lipotoxicity and accelerated catabolism. Clinically, this heterogeneity makes it difficult to predict which obese patients will develop these maladaptive responses (122).

#### 5.4.2 The central controversy: the “obesity paradox”

The foremost clinical controversy is the repeated observation of an “obesity paradox,” where overweight and moderately obese septic patients often exhibit lower mortality rates than their normal-weight counterparts. This starkly contradicts the expected pathophysiological harm, creating a significant challenge in prognostication and therapeutic strategy. The paradox is likely explained not by obesity being protective per se, but by confounding factors such as greater energetic reserves, differences in adipokine profiles, and the fact that BMI is a crude measure that does not differentiate between fat distribution, muscle mass, or the severity of underlying metabolic dysfunction. This paradox underscores a critical gap in our ability to risk-stratify septic patients based on simple anthropometrics and highlights the need for biomarkers that reflect functional adipose and immune health rather than mere mass (5).

In summary, the main clinical challenge lies in integrating the dual narrative of obesity in sepsis: it can be a source of pathological priming yet also associate with surprising resilience. This necessitates a shift from viewing obesity as a uniform risk factor toward personalized assessments that account for metabolic health, body composition, and functional immune-metabolic responses to tailor prognostication and therapy.

In conclusion, for the key pathways discussed in this review—such as the STING-ER Stress-mtROS Axis and the TGFβ-PDE3b-cAMP Axis—the core challenge for clinical translation lies in achieving ‘Tissue-Specific Regulation’. The unresolved key problem is how to inhibit pathological effects while preserving physiological function.

## 6 Conclusions and future research directions

### 6.1 Summary of core research findings

The adipo-immune-metabolic axis serves as a central regulatory hub for disease tolerance in sepsis. In this context, adipose tissue has evolved from a passive energy reservoir into an active “immunometabolic integration center.” Its functions are deeply aligned with three core hypotheses, constructing a host defense front through three primary roles:1. Strategic Energy Allocator: Embodying the “Metabolic Defense Priority” hypothesis, it releases large quantities of FFAs via lipolysis to fuel vital organs, converts them into efficient alternative fuels (e.g., β-hydroxybutyrate) via hepatic ketogenesis, and dissipates excess FFAs through BAT-UCP1-mediated thermogenesis. The core strategy is “protein sparing”—prioritizing the mobilization of renewable fat reserves to minimize the breakdown of skeletal muscle (especially respiratory muscles), thereby preserving structural and functional integrity. This is a key support for disease tolerance during the subacute phase of sepsis2. Active Immunological Buffer: Embodying the “Bidirectional Immunometabolic Crosstalk” hypothesis, immune cells within adipose tissue precisely regulate metabolism through sophisticated dialogue: M1 macrophages “accelerate” lipolysis via the IL-1β/ERK pathway to meet the acute phase energy crisis, while B cells provide a “braking” signal via the TGF-β/PDE3b/cAMP axis to prevent runaway lipolysis and lipotoxicity. Concurrently, adipocytes sense inflammatory signals via pattern recognition receptors and dynamically secrete adipokines (e.g., adiponectin, leptin) to buffer excessive inflammation and promote tissue repair, balancing defense efficiency with self-preservation.3.Dynamic System Adaptor: Embodying the “Stage-Specific Adaptation” hypothesis, the axis undergoes precise reprogramming along the septic course: dominance of skeletal muscle protein breakdown and initial lipolysis in the acute phase; peak lipolysis/ketogenesis and finely balanced immunometabolic crosstalk in the subacute phase; and lipolytic failure coupled with immune paralysis in the chronic phase, culminating in a vicious cycle of “metabolic exhaustion-immune dysregulation.” In summary, by integrating the three functions of “energy allocation - immunoregulation - stage-specific adaptation,” the adipo-immune-metabolic axis addresses the dual challenge of the “energy crisis + inflammatory storm” in sepsis. It provides core targets for moving beyond the limitations of traditional “pathogen resistance” therapy. Understanding and modulating the homeostatic operation of this axis is crucial for improving the high mortality rate and reducing the long-term burden of sepsis, meeting the clinical urgency for precise, targeted therapies.

### 6.2 Future research directions

To overcome current research bottlenecks, future efforts must focus on “filling mechanistic gaps, strengthening technological support, and bridging clinical needs” across three key dimensions to achieve a precise transition from basic research to clinical application:1. Cellular Dimension: Deciphering Heterogeneity, Ending Homogenized Research: Leverage single-cell multi-omics and spatial biology technologies to map the dynamic cellular landscape of the adipo-immune-metabolic axis across sepsis stages. This includes defining the spatiotemporal distribution and functional specialization of subsets like B1/B2/B10 cells and tissue-resident vs. monocyte-derived macrophages; elucidating depot-specific responses of subcutaneous vs. visceral white adipocytes; and uncovering how sepsis regulates the differentiation fate of adipose precursor cells. This will provide the basis for precisely targeting specific cell subtypes.2. Molecular Dimension: Exploring Non-Canonical Functions, Deciphering Pathway Crosstalk: Move beyond the “fuel role” of metabolites to deeply explore the “metabolite-epigenetics-immunity” regulatory network. Clarify the specific mechanisms by which metabolites like ketone bodies and Omega-3 fatty acids influence immune cell fate via HDAC inhibition and PPAR activation. Decipher the crosstalk mechanisms between the STING-ER Stress-mtROS Axis, the Insulin-INSR-Thermogenesis Axis, and the TGFβ-PDE3b-cAMP Axis, identifying molecular switches (e.g., mtROS threshold, CHOP expression level) that govern pathway transitions3. Systemic Dimension: Deciphering Inter-organ Communication, Accounting for Comorbidities: Investigate the remote regulatory mechanisms mediated by adipose tissue via extracellular vesicles, defining how molecules carried by EVs (e.g., STING-activating peptides, microRNAs) modulate the immunometabolic state of distant organs like the liver and heart. Prioritize understanding the impact of comorbidities (e.g., obesity, diabetes) on axis function, analyzing the unique mechanisms underlying excessive/impaired lipolysis and exacerbated lipotoxicity in these patients. This will lay the groundwork for developing personalized intervention strategies for special populations.

This research requires reliance on novel models such as humanized adipose tissue-transplanted mice and sepsis organoids to circumvent the species-specific limitations of traditional animal models and ensure the clinical relevance and translatability of the findings.

### 6.3 Clinical translation

Future clinical translation must adhere to the principles of “mechanism-guided, stage-adapted, and precision-individualized.” To address the translational barriers previously discussed, a comprehensive “Stratification-Intervention-Validation” system should be established:1. Develop Integrated Biomarker Panels for Precision Stratification: Move beyond reliance on single biomarkers. Develop integrated panels combining metabolites, immune cell phenotypes, and clinical features—based on lipidomics, immunophenotyping, and clinical parameters—and validate their value for patient stratification and prognosis prediction through multi-center clinical trials. Simultaneously, leverage AI algorithms to integrate these indicators and create bedside rapid staging tools, addressing the critical challenge of difficult disease phase determination2. Implement Dynamic, Precision Interventions Guided by Stage-Specificity: Adhere to the principle of stage-specific adaptation to administer dynamic, precise interventions aimed at breaking the vicious cycle of “metabolic exhaustion-immune paralysis.”3. Expand the Diversified Therapeutic Arsenal to Overcome Target Translation Bottlenecks: Explore the immunometabolic regulatory potential of existing drugs—Metformin improves adipocyte insulin resistance via AMPK activation; PPARγ agonists (e.g., Rosiglitazone) inhibit lipotoxicity, showing promise for septic patients with comorbidities. Furthermore, advance research on novel therapeutics: develop tissue-specific agents targeting core nodes like PDE3b, UCP1, and STING (e.g., BAT-targeted UCP1 agonists to avoid cardiovascular side effects); and promote the clinical validation of adipokine recombinant proteins (e.g., CTRP9) for conditions like septic cardiomyopathy.

In summary, deciphering the complexity of the septic adipo-immune-metabolic axis necessitates close collaboration between basic scientists and clinicians. By integrating cutting-edge technologies to deepen mechanistic understanding and using these insights to design innovative clinical translation strategies, we can aspire to achieve the overarching goal of improving sepsis outcomes by enhancing the patient’s own disease tolerance capacity. Successful clinical translation is inseparable from tight basic-clinical collaboration: it requires establishing a “Sepsis Immunometabolic Database” that integrates clinical, imaging, and omics data from multi-center patients to continuously refine stratification standards and intervention protocols. Concurrently, attention to sex and age differences is paramount, developing subgroup-specific strategies for women (considering estrogen/ERα signaling) and the elderly (considering immunometabolic aging), ultimately realizing the core objective of “enhancing disease tolerance and improving sepsis outcomes.
